# Correction and removal of expression of concern: Palladium supported on mixed-metal–organic framework (Co–Mn-MOF-74) for efficient catalytic oxidation of CO

**DOI:** 10.1039/d4ra90078b

**Published:** 2024-07-22

**Authors:** Reda S. Salama, Mohammed A. Mannaa, Hatem M. Altass, Amr Awad Ibrahim, Abd El-Rahman S. Khder

**Affiliations:** a Basic Science Department, Faculty of Engineering, Delta University for Science and Technology Gamasa Egypt reda.salama@deltauniv.edu.eg dr.reda.salama@gmail.com; b Chemistry Department, Faculty of Science, Sa'ada University Yemen mnnaam@yahoo.com; c Research Laboratories Unit, Chemistry Department, Faculty of Applied Science, Umm Al-Qura University 21955 Makkah Saudi Arabia hutass@uqu.edu.sa; d Chemistry Department, Faculty of Science, Mansoura University Mansoura Egypt amr_awad@mans.edu.eg askhder2244@yahoo.com

## Abstract

Correction and removal of expression of concern for ‘Palladium supported on mixed-metal–organic framework (Co–Mn-MOF-74) for efficient catalytic oxidation of CO’ by Reda S. Salama *et al.*, *RSC Adv.*, 2021, **11**, 4318–4326, https://doi.org/10.1039/D0RA09970H.

The authors regret that the original XRD measurements and figures had a high signal-to-noise ratio, which resulted in excessive smoothing of the data. The authors have now repeated the experiments, and have provided the replacement figures here:
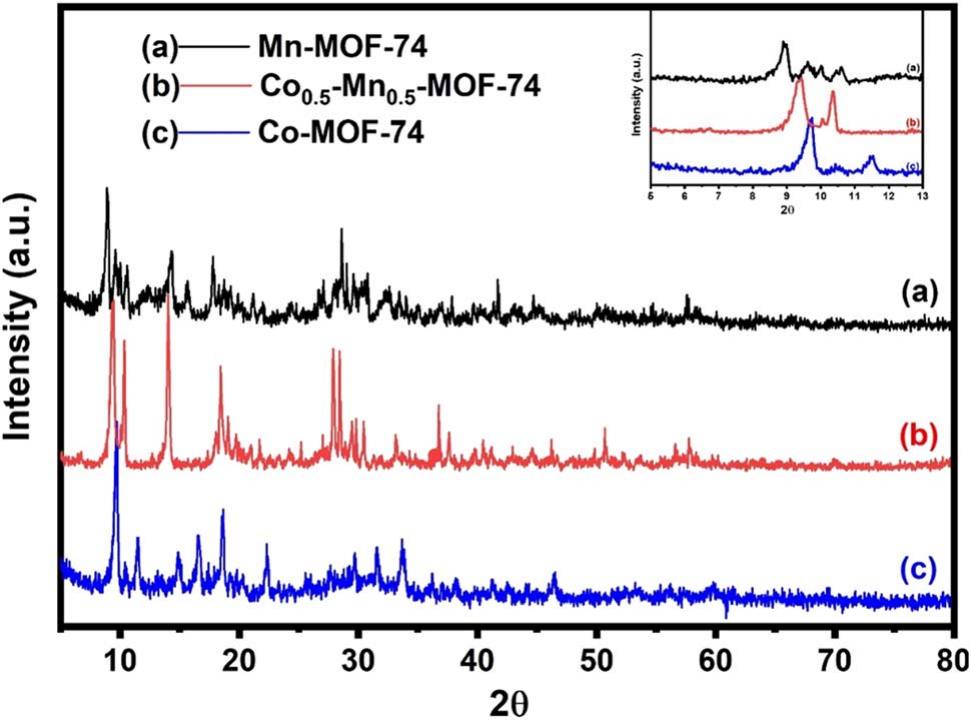


**Fig. 1** XRD patterns of monometallic and bimetallic MOF-74.
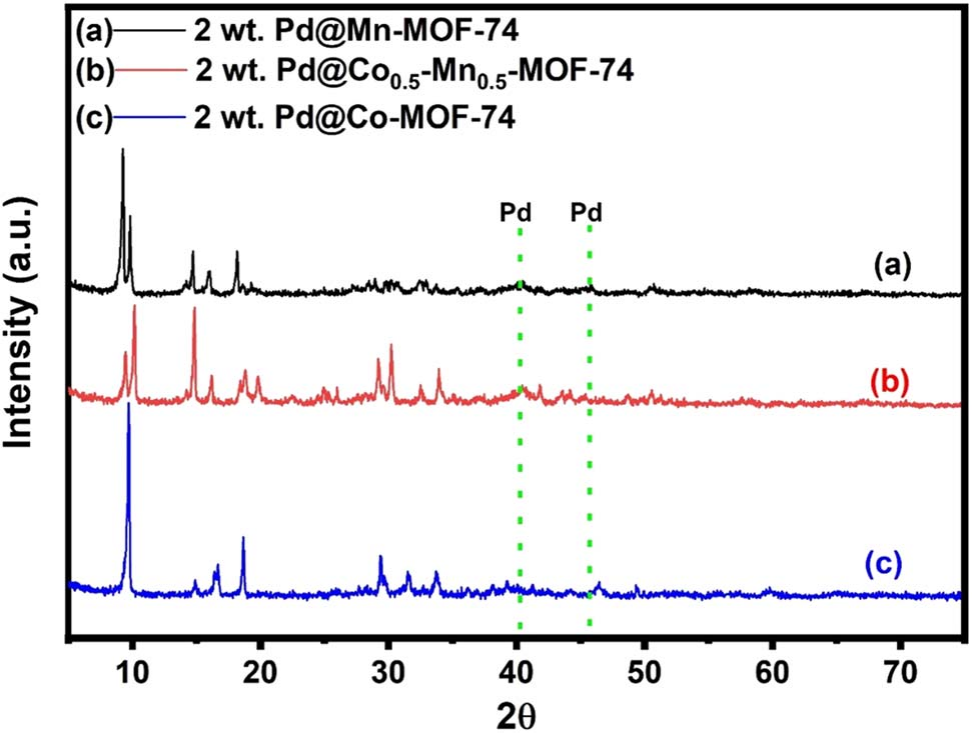


**Fig. 2** XRD patterns of 2 wt% Pd NPs loaded on (a) Mn-MOF-74, (b) Co_0.5_-Mn_0.5_-MOF-74 and (c) Co-MOF-74.

The measurements for peaks at 2*θ* are adjusted slightly, and the text in section **3.1 X-ray diffraction patter (XRD)** should be:

Mn-MOF-74 shows characteristic peaks at 2*θ* equal 8.5, 9.6, 10.4, 11.9, 14.2, 15.5, 17.9, 19.6, 21.2, 27.1, 28.6, 30.6, 32.5, 37.6, 41.7, 54.3, and 57.5°. On the other hand, XRD patterns of Co-MOF-74 were recognized at 2*θ* equal 9.5, 10.2, 11.4, 14.7, 16.4, 18.4, 26.8, 29.5, 31.6, 33.4, 37.8, 46.5, and 59.6°, which were almost identical to the previously reported XRD pattern of Mn-MOF-74,^41–43^ and Co-MOF-74,^44–46^ respectively.

An independent expert has viewed the corrected images and raw data and has concluded that they are consistent with the discussions and conclusions presented.

This correction supersedes the information provided in the Expression of Concern related to this article.

The Royal Society of Chemistry apologises for these errors and any consequent inconvenience to authors and readers.

